# A 6-year nationwide population-based study on the current status of gastric endoscopic resection in Korea using administrative data

**DOI:** 10.1038/s41598-023-34215-7

**Published:** 2023-05-03

**Authors:** Jae Yong Park, Mi-Sook Kim, Beom Jin Kim, Jae Gyu Kim

**Affiliations:** 1 Department of Internal Medicine, Chung-Ang University College of Medicine, 84 Heukseok-ro, Dongjak-gu, Seoul, 06974 Republic of Korea; 2 Biomedical Research Institute, Chung-Ang University Hospital, Seoul, Republic of Korea; 3 Division of Clinical Epidemiology, Medical Research Collaborating Center, Biomedical Research Institution, Seoul National University Hospital, Seoul, Republic of Korea

**Keywords:** Gastric cancer, Oesophagogastroscopy

## Abstract

Gastric endoscopic resection (ER) is widely performed in Korea. This study aimed to investigate the overall status of gastric ER in Korea. We enrolled ESD or EMR cases performed for gastric cancer and adenoma from 2012 to 2017 by searching the NHIS database. The annual trend of gastric ER and the clinical characteristics were investigated. Institutions were classified into very high-, high-, low-, and very low volume centers (VHVC, HVC, LVC, and VLVC) by the procedure numbers, and institutional types, regional distributions, and medical resources were investigated accordingly. There were 175,370 ER cases during the study period, with an increasing trend over time. The average annual ESD procedure numbers were 3.9, 54.5, 249.5, and 540.3 cases in 131 VLVCs, 119 LVCs, 24 HVCs, and 12 VHVCs, respectively. Among ESD-performing institutions, 44.8% were located in the Seoul Capital Area. The distribution of medical resources showed a positive correlation with the procedural volume. Similar tendencies were also demonstrated in EMR, with some differences in hospital types and regional distribution. Gastric ER and ESD are increasing in Korea. There was a significant variance in the number of ER procedures and the distribution of types, regions, and medical resources according to the procedural volume.

## Introduction

With recent developments in endoscopic devices and techniques, endoscopic resection (ER) has become widely adopted to treat superficial gastric neoplastic lesions^1^. Studies on the long- and short-term results of endoscopic submucosal dissection (ESD) have shown that ESD is comparable to surgery for the treatment of early gastric cancer (EGC) with specific indications^2,3^. Currently, there is a consensus that ESD is the optimal treatment for differentiated-type mucosal cancers of the stomach^4^. Furthermore, guidelines also recommend ER for visible gastric adenoma as a precancerous lesion^5,6^.

In Korea, the National Cancer Screening Program is in operation, which includes gastric cancer screening for adults aged 40 years or more via upper gastrointestinal (GI) series or endoscopic examinations biennially^7^. As a result, a significant proportion of newly discovered gastric cancers are now identified in their early stages, and the detection rate of gastric precancerous lesions is also high^8^. Consequently, this has led to an increase in gastric lesions corresponding to the indications for ER, contributing to the improved clinical outcome and prognosis of gastric cancer^9^. In line with these changes, ESD has been covered by the National Health Insurance (NHI) since November 2011 in Korea. As one of the countries where gastric ER is most actively performed, EGC and gastric adenoma are now commonly treated with ER in Korea.

Since ER is performed in many institutions nationwide in Korea, extensive research using national medical data is essential to comprehensively understand the current status of the procedure. Furthermore, this will also be critical systematic data that could be used to manage and improve the quality of gastric ER. Previously, Kim et al. reported the ESD status of EGC in Korea between 2011 and 2014 using NHI claims data^1^. However, ever since, there has never been a large-scale nationwide investigation into the situation of gastric ER. Furthermore, most studies have been limited to ESD performed on EGC only.

Therefore, a study on the current status of gastric ER, including ESD and endoscopic mucosal resection (EMR) for gastric cancer and adenoma, at a national level is crucial. This study aimed to investigate the overall status of gastric ESD and EMR in Korea for 6 years, from 2012 to 2017, using the National Health Insurance Service (NHIS) database. This study will provide us with valuable and comprehensive data, including annual trends, procedure characteristics, and regional and resource distribution by type of institution and procedural volume, which could be used for understanding the status of gastric ER in Korea.

## Methods

### Data sources

The customized database from the NHIS database’s data-sharing service was used for the study. We extracted data from the database from January 1, 2012, to December 31, 2017. Since the NHIS is a mandatory universal health insurance system, Korean citizens are obliged to subscribe to health insurance and pay the premiums according to the insurance subscriber category to which they belong. This system consists of two health care programs, NHI and Medical Aid, where NHI covers 97% of the Korean population, and Medical Aid program covers the remaining 3%. This administrative database is also used for billing medical expenses. Notably, this database provides anonymized data on the qualification, statement, treatment details, type of disease, prescription details, and information of clinics. Therefore, research using the NHIS database is advantageous for comprehensively understanding the clinical situation on a nationwide scale.

### Study design and selection of target population

We enrolled ESD or EMR cases performed for gastric cancer and adenoma from January 2012 to December 2017 since coverage of ESD by the NHI was implemented in November 2011. We searched the NHIS database by combining diagnostic and procedure codes to identify the target cases. Cases were included in this study if a procedure code for upper GI ESD or EMR was present in the claims data and diagnostic codes for gastric cancer or adenoma were present at the time of the procedure. The cases were excluded if qualification data were missing.

Diagnostic codes (ICD-10th codes) included C16 (C16.0, C16.1, C16.2, C16.3, C16.4, C16.5, C16.6, C16.8, C16.9) for gastric cancer, and D00.2, D13.1, and D37.1 for gastric adenoma. Cases were included when these diagnostic codes were designated as the primary diagnosis or one of the first 4 secondary diagnoses. Procedure codes for EMR and ESD included QZ933, Q7652, QX704, and QX701. To further distinguish between ESD and EMR, a combination of procedure codes and codes for treatment materials was used. ESD was defined by the following procedure codes: QZ933, QX704, or QX701. Additionally, cases charged with the codes for EMR (Q7652), simultaneously with material codes for ESD knives, were also defined as ESD. This was due to the notice that ESD performed in a piecemeal resection manner should be charged as EMR, according to the reimbursement criteria based on the Regulation for Criteria for Providing Reimbursed Services in the NHI. EMR was defined as the remnant cases with the procedure code Q7652, not classified as ESD cases.

The algorithm mentioned above for identifying target cases was validated by retrospective medical record analysis of an individual medical institution. To do so, we compared the case identification results with the algorithm from electronic medical record data with a reference standard (chart-based diagnosis). This study was approved by the Institutional Review Board of Chung-Ang University Hospital (IRB No. 1772-001-290) and conducted in accordance with the Declaration of Helsinki. Patient consent was waived, given that the NHIS database is a publicly available anonymized dataset.

### Study variables and categorization

The demographic information and clinical characteristics of the enrolled patients, including age, sex, socioeconomic status (SES), type of gastric disease, and residence, as well as the characteristics of the hospitals, were analyzed. Additionally, procedural volume, regional distribution, and SES were categorized for analysis.

The procedural volume was expressed as the number of cases during the study period and was initially evaluated as a continuous variable. Using the number of ESD and EMR procedures performed by individual institutions during the study period, the procedural volume was classified as follows: if the mean annual number of procedures was less than 10, it was classified as a very low volume center (VLVC). The remaining institutions were classified into very high-, high-, and low-volume- institutions (VHVC, HVC, and LVC), respectively, in the order from the highest to the lowest procedure numbers during the study period, so that the cumulative sum of the procedure numbers in each category could be evenly distributed. When performing subgroup analysis for ESD or EMR each, only the corresponding procedures were counted and classified in the same manner as described above. The region was divided into three categories: The Seoul Capital Area (Seoul, Incheon, and Gyeonggi Province), the metropolitan city, and the province. For classification by SES, the top 25%, bottom 25%, and middle 50% of the population eligible for NHI were divided based on income level, and the recipients in Medical Aid Program were classified separately.

### Statistical analysis

The differences in the demographic and clinical characteristics between the groups were tested using the chi-square test for categorical variables and the t-test or Wilcoxon rank sum test for continuous variables. The generalized estimating equation was used to consider the patients who undergo multiple ER procedures. Bowker's test of symmetry was used to determine the concordance between the location of patients’ residences and the medical institution where they received ER. All tests were 2-sided with a P value less than 0.05 indicating statistical significance. Statistical analyses were performed using SAS Enterprise Guide 7.1 (SAS Institute INC., Cary, NC, USA).

## Results

### The annual trend of ER procedures

**Figure 1:**
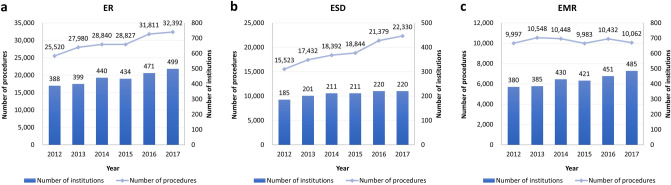
The total annual number of ER, ESD, and EMR procedures and operating institutions. (**a**) The numbers of ER (line) and the institutions performing the procedures (bar) steadily increased during the study period. (**b**) The numbers of ESD (line) and the institutions conducting the procedures (bar) steadily increased during the study period. (**c**) The numbers of EMR (line) did not show meaningful change, while the institutions performing the procedures (bar) increased during the study period. *ER* endoscopic resection, *ESD* endoscopic submucosal dissection, *EMR* endoscopic mucosal resection.

From 2012 to 2017, a total of 175,370 cases of ER were performed in 855 institutions. The number of ER increased by 26.9% from 25,520 in 2012 to 32,392 in 2017, and the number of institutions increased by 28.6% from 388 to 499 during the same period (Fig. 1a). When ESD and EMR were analyzed separately, a total of 113,900 cases of ESD were performed in 286 institutions. The number of ESD increased by 43.9% from 15,523 in 2012 to 22,330 in 2017, and the number of institutions increased by 18.9% from 185 to 220 during the same period (Fig. 1b). Meanwhile, a total of 61,470 cases of EMR were performed in 838 institutions. The number of EMRs did not significantly change during the study period, while the number of institutions increased by 27.6% from 380 to 485 (Fig. 1c). Furthermore, in 2012, 60.8% of ER cases were ESDs, which increased to 68.9% in 2017.

The subgroup analysis by disease revealed that the number of ER and ESD steadily increased for both gastric cancer and adenoma, and this trend was not evident for EMR (Supplementary Table S1). Notably, gastric cancer was mainly treated with ESD rather than EMR, and the proportion of gastric cancer treated with ESD increased over time.

### The baseline characteristics and distribution of ER by type of institution

When dividing the 855 institutions by type, there were 319 clinics, 228 hospitals, 265 general hospitals, and 43 tertiary hospitals. The average annual ER procedures performed in each group showed significant differences, with case values reported as 3.2 (± 7.2), 5.2 (± 9.3), 40.4 (± 74.1), and 411.2 (± 281.2), respectively (Table 1). Among all the institutions performing ER, only 6.9% (22/319) of the clinics and 20.2% (46/228) of the hospitals performed ESD, whereas 66.0% (175/265) of the general hospitals and all (43/43) of the tertiary hospitals performed ESD. The median annual procedural volume of ER in clinics and hospitals was 1.5 and 2 cases, respectively. Furthermore, the gap between the minimum and the maximum number was extensive, even among the same type of institutions. When ESD and EMR were analyzed separately, there was also a large deviation in the procedural volume among the same types of institutions, except for clinics (Supplementary Tables S2, S3). The results of subgroup analyses according to procedure type (ER, ESD, and EMR) and disease (gastric cancer and adenoma) are described in Supplementary Tables S4–S9.

**Table 1:** The average annual number of ER by type of medical institution.

Average annual number of procedures	Total (n = 855)	Type of institution			
		Clinic (n = 319)	Hospital (n = 228)	General hospital (n = 265)	Tertiary hospital (n = 43)
Mean ± SD	35.8 ± 115.7	3.2 ± 7.2	5.2 ± 9.3	40.4 ± 74.1	411.2 ± 281.2
Median (IQR)	2.5 (1.0, 11.3)	1.5 (1.0, 2.7)	2.0 (1.0, 4.0)	11.2 (3.0, 40.2)	348.5 (243.3, 483.5)
Min	1.0	1.0	1.0	1.0	63.0
Max	1641.2	89.3	55.5	637.3	1,641.2

*ER* endoscopic resection, *SD* standard deviation, *IQR* interquartile range.

The baseline demographics of the included population are shown by type of institution in Table 2. The mean age, sex, SES, type of gastric disease, and location of residence significantly differed among the groups. The proportion of the high SES group was larger in tertiary hospitals than in other institutional types. Notably, the proportion of gastric cancer steadily increased from the clinic (2.0%) to the tertiary hospital (46.0%).

**Table 2:** The baseline characteristics of the included patients by type of medical institution.

Variables	Total (n = 168,187)	Type of institution				p-value*
		Clinic (n = 3365)	Hospital (n = 4651)	General hospital (n = 59,653)	Tertiary hospital (n = 100,518)	
Age, mean ± SD	64.5 ± 10.5	59.8 ± 12.2	62.5 ± 11.0	64.8 ± 10.5	64.5 ± 10.3	0.011
Sex, n (%)						< 0.001
Male	112,523 (66.9)	1616 (48.0)	2750 (59.1)	39,549 (66.3)	68,608 (68.3)	
Female	55,664 (33.1)	1749 (52.0)	1901 (40.9)	20,104 (33.7)	31,910 (31.8)	
SES						< 0.001
Top 25%	66,162 (39.3)	1226 (36.4)	1614 (34.7)	21,666 (36.3)	41,656 (41.4)	
Middle 50%	65,321 (38.8)	1364 (40.5)	1870 (40.2)	23,802 (39.9)	38,285 (38.1)	
Bottom 25%	26,704 (15.9)	582 (17.3)	864 (18.6)	9843 (16.5)	15,415 (15.3)	
Medical aid	6445 (3.8)	111 (3.3)	219 (4.7)	3101 (5.2)	3,014 (3.0)	
Missing	3555 (2.1)	82 (2.4)	84 (1.8)	1241 (2.1)	2,148 (2.1)	
Gastric disease						< 0.001
Cancer	65,298 (38.8)	68 (2.0)	499 (10.7)	18,491 (31.0)	46,240 (46.0)	
Adenoma	102,889 (61.2)	3297 (98.0)	4152 (89.3)	41,162 (69.0)	54,278 (54.0)	
Residence						< 0.001
Capital region	68,542 (40.8)	1208 (35.9)	1982 (42.6)	23,497 (39.4)	41,855 (41.7)	
Metropolitan City	37,670 (22.4)	1036 (30.8)	1172 (25.2)	13,094 (22.0)	22,368 (22.3)	
Province	61,945 (36.8)	1121 (33.3)	1496 (32.2)	23,052 (38.7)	36,276 (36.1)	
Missing	30 (0.0)	0 (0.0)	1 (0.0)	10 (0.0)	19 (0.0)	

*SD* standard deviation, *SES* socioeconomic status.

*To consider the patients who underwent multiple procedures, the difference in the distribution of medical institution types according to demographic and clinical characteristics was tested using the generalized estimating equation.

### The average annual number of ESD and EMR by procedural volume

**Figure 2:**
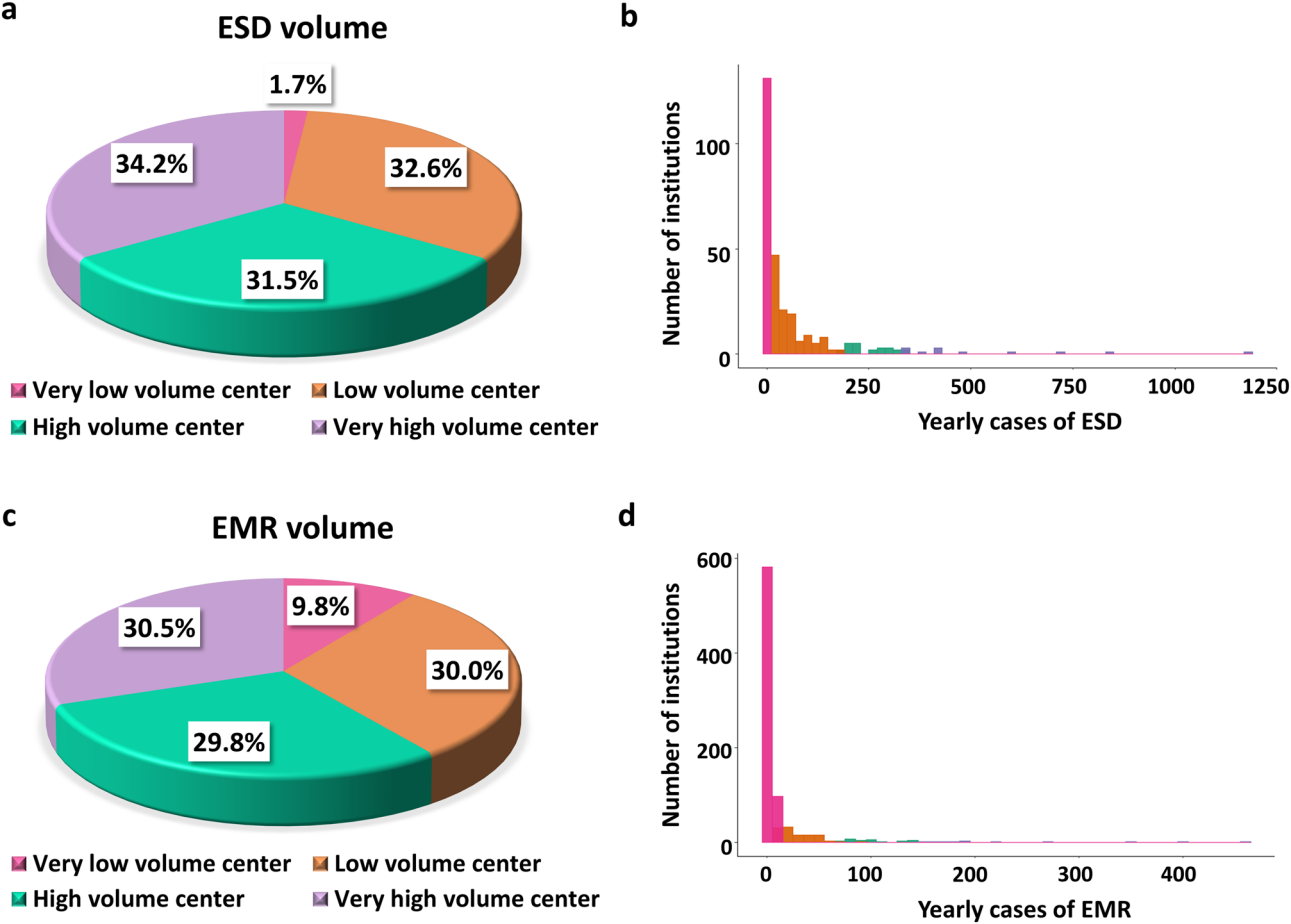
Distribution of the number of procedures and institutions by procedural volume. (**a**) The number of ESD performed in each group is proportional to the total number of ESD procedures. (**b**) The distribution of institutions is presented according to the annual procedural volume of ESD. (**c**) The number of EMR performed in each group is proportional to the total number of EMR procedures. (**d**) The distribution of institutions is presented according to the annual procedural volume of EMR. *ESD* endoscopic submucosal dissection, *EMR* endoscopic mucosal resection.

When dividing the 286 institutions by procedural volume of ESD, there were 131 VLVCs, 119 LVCs, 24 HVCs, and 12 VHVCs. Each group's average annual number of ESD procedures showed significant differences (Table 3). While VLVCs accounted for 45.8% of the total number of institutions, the procedures performed in this group only accounted for 1.7% of the total ESD procedures (Fig. 2a). On the other hand, 65.7% of the ESDs were performed in VHVCs and HVCs, which accounted for only 12.6% of the total number of institutions (Fig. 2b). The volume classification and procedural distribution of EMR are also demonstrated in Supplementary Table S10. For EMR, only 9.8% of the total procedures were performed in VLVCs, which accounted for 80.9% of the total institutions (Fig. 2c). On the other hand, 60.3% of the total procedures were performed in VHVCs and HVCs, which accounted for only 5.1% of the total institutions (Fig. 2d).

**Table 3:** The average annual number of ESD by procedural volume.

Average annual number of procedures	Total (n = 286)	Procedural volume of ESD			
		Very low (n = 131)	Low (n = 119)	High (n = 24)	Very high (n = 12)
Mean ± SD	68.1 ± 133.1	4.1 ± 2.8	54.8 ± 43.6	249.5 ± 51.0	540.3 ± 258.6
Median (IQR)	13.3 (4.0, 64.5)	3.3 (1.0, 6.2)	39.7 (19.7, 74.0)	238.4 (200.3, 293.3)	421.7 (362.4, 664.9)
Min	1.0	1.0	10.7	186.8	336.2
Max	1176.5	9.8	180.8	334.8	1176.5

*ESD* endoscopic submucosal dissection, *SD* standard deviation, *IQR* interquartile range.

### Distribution of institutional type by procedural volume

The distribution of institutional type was investigated according to the classification by procedural volume of ESD. Notably, the VLVC group was comprised of clinics (16.8%), hospitals (25.2%), and general hospitals (58.0%), but not tertiary hospitals (Table 4). All clinics were included in the VLVC group, and all hospitals were included in the VLVC or LVC groups. The HVC group was composed of general hospitals (25.0%) and tertiary hospitals (75.0%), and tertiary hospitals accounted for the absolute majority (91.7%) of the VHVC group. The distribution of institutional type by procedural volume of EMR is shown in Supplementary Table S11.

**Table 4:** Distribution of institutional type according to the classification by procedural volume of ESD.

Type of institution	Procedural volume of ESD			
	Very low (n = 131)	Low (n = 119)	High (n = 24)	Very high (n = 12)
Clinics	22 (16.8%)	0 (0.0%)	0 (0.0%)	0 (0.0%)
Hospitals	33 (25.2%)	13 (10.9%)	0 (0.0%)	0 (0.0%)
General hospitals	76 (58.0%)	92 (77.3%)	6 (25.0%)	1 (8.3%)
Tertiary hospitals	0 (0.0%)	14 (11.8%)	18 (75.0%)	11 (91.7%)

*ESD* endoscopic submucosal dissection.

### Regional distribution of institutions by procedural volume

Among the total institutions performing ESD, 44.8% (128/286) were distributed in the Seoul Capital Area, 23.4% (67/286) in metropolitan cities, and 31.8% (91/286) in provinces. The regional distribution of institutions was further investigated according to the classification by procedural volume (Table 5). For VLVCs, 35.9% were distributed in the Seoul Capital Area, 25.2% in metropolitan cities, and 38.9% in provinces. In cases of LVCs and HVCs, the proportions for each regional classification were 53.8%, 19.3%, and 26.9%, and 41.7%, 29.2%, and 29.2%, respectively. However, all but one (91.6%) of the VHVCs were located in the Seoul Capital Area or metropolitan cities. The regional distribution of institutions by procedural volume of EMR is shown in Supplementary Table S12.

**Table 5:** Regional distribution of institutions according to the classification by procedural volume of ESD.

Region	Procedural volume of ESD			
	Very low (n = 131)	Low (n = 119)	High (n = 24)	Very high (n = 12)
The Seoul Capital Area	47 (35.9%)	64 (53.8%)	10 (41.7%)	7 (58.3%)
Metropolitan cities	33 (25.2%)	23 (19.3%)	7 (29.2%)	4 (33.3%)
Provinces	51 (38.9%)	32 (26.9%)	7 (29.2%)	1 (8.3%)

*ESD* endoscopic submucosal dissection.

Considering the migration of patients when choosing the institutions, we performed additional analysis by comparing the patient’s residence and the location of the institution where the procedure was performed. Among the residents outside of the Seoul Capital Area, 23.0% (13,963/60,600) underwent ESD, and 12.7% (4,956/39,015) underwent EMR in the Seoul Capital Area, respectively (Supplementary Tables S13, S14). In cases of the residents of provinces, 54.6% (21,585/39,506) underwent ESD, and 48.4% (10,869/22,439) underwent EMR in the Seoul Capital Area or Metropolitan cities, respectively. Meanwhile, more than 95% of residents in the Seoul Capital Area received ESD or EMR in the same area.

### Distribution of medical resources by procedural volume

The distribution of medical resources was investigated according to the classification by procedural volume of ESD and EMR. The number of doctors in each group significantly increased in ascending order, from VLVC to VHVC group, for both procedures (Table 6, Supplementary Table S15). Furthermore, the number of specialists, hospital beds, and operating rooms also showed a similar tendency of positive correlation with the procedural volume.

**Table 6:** Distribution of medical resources according to the classification by procedural volume of ESD.

Medical resources	Procedural volume of ESD			
	Very low (n = 131)	Low (n = 119)	High (n = 24)	Very high (n = 12)
Doctors	28.2 ± 25.9	140.4 ± 121.6	358.2 ± 108.5	765.3 ± 495.3
Specialists	24.4 ± 19.0	88.7 ± 60.0	201.2 ± 68.8	434.1 ± 270.6
Hospital beds	197.6 ± 128.1	429.6 ± 201.2	800.9 ± 177.3	1259.6 ± 573.1
Operating rooms	3.1 ± 2.3	7.6 ± 5.1	16.3 ± 7.8	33.3 ± 20.1

Values are mean ± standard deviation unless stated otherwise.

*ESD* endoscopic submucosal dissection.

## Discussion

This nationwide, population-based study was designed to investigate the current status of ESD and EMR for EGC and gastric adenoma in Korea over 6 years. Our study demonstrated the annual trends of ER procedures, differences in the procedure numbers according to hospital types, and distribution of institutions and medical resources, including regional distributions according to procedural volumes.

From 2012 to 2017, ER for EGC and gastric adenoma, as well as the number of institutions performing these procedures, steadily increased. Over the 6 years, the proportion of ESD among total ER procedures increased from 60.8 to 68.9%, indicating that more cases are being treated through ESD. Indeed, the number of ESD and institutions increased by 43.9% and 18.9%, respectively. On the other hand, the number of EMR showed insignificant change, although the number of institutions increased by 27.6% over the same period. In particular, ESD accounted for about 80% of endoscopically treated EGC. For gastric adenoma, the proportion of ESD also steadily increased to 60.3% over time. These observed trends show that more institutions are performing ER, and the increase in ER is mainly due to the rise in ESD. Notably, ESD was the most preferred treatment option for endoscopically treated EGCs, and the application of ESD to gastric adenoma was also noted as increasing in Korea. During the whole study period, ESD for gastric adenoma was also covered by NHIS if the resected tissues were larger than 3 cm in size, which might be one of the reasons for the active performance of ESD for gastric adenoma.

Compared to EMR, ESD allows more precise control of the extent and depth of the resection. Since the resection can be done by directly viewing the dissection plane, and fibrotic lesions can be meticulously managed, ESD is considered more appropriate for securing the resection margin^10,11^. This is an important issue, especially in the case of gastric cancer, as a negative resection margin is the core factor of curative resection. Indeed, the local recurrence rate of EGC is lower in ESD than in EMR^12,13^. There is also an issue that discrepancies often exist between pre-and post-resection pathologic diagnosis. In up to 11–26% of cases, adenoma is later determined to be EGC after the ESD, suggesting that ESD might be an optimal option for both diagnosis and curative treatment, even for some adenoma cases^14,15^.

There might be some explanations for the increase in ESD procedures for EGC over time. The National cancer screening program for gastric cancer was implemented in 2002 in Korea, and the participation rate steadily increased from 28.0% in 2007 to 51.9% in 2016^16^. Thanks to this national initiative, early detection of gastric cancer has increased over time, despite the decreasing rate of gastric cancer in Korea^17,18^. According to a Korean registry of surgically treated gastric cancers, the proportion of EGC consistently increased from 57.7% in 2009 to 63.6% in 2019^18^. In addition, according to the results from adequacy evaluation of stomach cancer by the Health Insurance Review and Assessment Service in Korea, the proportion of surgery for stage IA gastric cancer steadily decreased from 54.6% in 2014 to 50.3% in 2017. In the meanwhile, the proportion of endoscopic resection continuously increased from 45.4 to 49.7%^19,20^, indicating that endoscopic treatment for EGC has been being more actively performed over time. Accumulation of favorable long-term outcome data in patients with expanded indications might also have affected this trend^21,22^. Thus, early detection of gastric cancer and increasing application of ESD for EGC could be both accountable for the increment in ESD for gastric cancer.

About two-thirds of the patients were male, reflecting the male predilection for gastric cancer or adenoma^17^. Cancer patients were more likely to visit higher-level institutions compared to those with adenoma, which seems plausible considering that multidisciplinary approach and specialized experience is crucial for the management of cancer. Previous studies have shown similar findings that patients with cancer were more inclined to visit hospitals than clinics to seek out cancer specialists^23,24^. Patients visiting higher-level institutions were also older. This finding might be due to the fact that aging is undeniably a major risk factor for cancer^25^, and higher proportion of cancer patients were included in the higher-level institution group. In addition, elderly patients are generally at greater risk to have multiple comorbidities and poor general conditions, increasing the procedure-related risks, which might also be a reason for visiting these institutions^26,27^. There were also differences in the institutional distribution according to the SES and the location of residence, which may be related to higher healthcare costs in higher-level institutions. Therefore, these findings reflect differences in the selection and access to healthcare institutions according to various clinical and socioeconomic factors.

There were differences in the procedural volume according to the type of institution. The median annual number of ER for clinics and hospitals was generally very small, whereas that of tertiary hospitals was overwhelmingly large, more than 10 times higher than that in general hospitals. In addition, a considerable deviation existed in the procedural numbers even among the same type of institutions. Interestingly, while the proportion of clinics was the highest among the institutions performing EMR, clinics only accounted for 7.7% of the institutions implementing ESD. Additionally, the distribution of ESD procedures was investigated depending on the procedural volume. While the VLVC group accounted for nearly half of the total institutions, only 1.8% of the total ESDs were performed in this group. On the contrary, 2/3 of the total ESDs were performed in the VHVC and HVC groups, which accounted for 12.9% of the total institutions. Meanwhile, most of the VHVCs were tertiary hospitals, but vice versa was not always the case. In brief, there was a tendency of a positive correlation between the type of institutions and the procedural volume, although there were exceptions depending on individual institutions. More importantly, it was noticeable that ESD procedures were concentrated mainly in some large-volume institutions. A similar tendency was also observed in gastric EMR. The proportion of VLVCs was even higher in EMR than in ESD, implying that the procedural volume was usually minimal in most EMR-performing institutions, unlike ESD. This is likely due to the differences in technical difficulties, required experience level, and necessary facilities between ESD and EMR. The technical challenges of EMR are lower than that of ESD, making EMR relatively easier to implement at a lower-level institution without heavy resources, such as clinics and hospitals^28,29^. On the other hand, ESD is not only technically demanding but also has a risk of complications, sometimes requiring hospitalization, transfusion, and emergency radiologic or surgical interventions. Consequently, these challenges make it difficult to implement gastric ESD in small facilities^28,30^. Lack of expertise in cancer treatment and a multidisciplinary cooperative system could be another barrier to performing ESD in relatively small institutions, leaving relatively few ESD-performing institutions with minimal procedural volumes.

The analysis of regional distribution demonstrated that approximately 2/3 of ESD-performing institutions were located in the Seoul Capital Area and metropolitan cities, where 91.7% of VHVCs were concentrated. Although this tendency was similar for EMR, the concentration of VHVC institutions in the Seoul Capital Area was less distinct for EMR than ESD. Interestingly, about half of provincial residents underwent ER in the Seoul Capital Area or metropolitan cities, while the proportion was higher for ESD. Among the residents outside the Seoul Capital Area, the proportion of patients visiting the institutions in the Seoul Capital Area was nearly twice as high in ESD compared to EMR. This finding infers that many residents in rural areas tend to receive gastric ER in large cities, especially in the case of ESD. Notably, Korea's whole country is a daily living area, which might partially explain why high-volume institutions and procedures are concentrated in large cities. Likewise, the higher proportion of patients with gastric cancer among ESD recipients may be another reason for this difference.

Our study has some limitations. Firstly, there was an inherent limitation of administrative data. Since we could not confirm the characteristics of the gastric lesion due to the lack of this information in the dataset, differences in these characteristics could not be included in the analyses. Also, there may have been some under- or overestimation when identifying the cases of gastric cancer or adenoma using the diagnostic codes for the same reason. Nevertheless, we tried to minimize this error by going through the validation of the algorithm for case selection. Secondly, the analysis did not consider the learning curve effect or procedural volume of individual operators. These factors could also be associated with the volume-outcome association. Thirdly, we did not include analyses on hospital stays or medical costs. Since the main scope of this study was the current overall status and distribution by type and volume of institutions, procedural outcomes were not covered. Instead, this topic will be investigated in an ongoing study that we are conducting. Nevertheless, economic analysis could be give us valuable insight in terms of cost-effectiveness and resource distribution, which warrants attention and further research.

To our knowledge, this is the first nationwide study on the current status of ER, including ESD and EMR, for gastric cancer and adenoma in Korea using the NHIS database. Additionally, it has the strength of analyzing long-term data over 6 years and comparing the distributions and characteristics by type and procedural volume of the medical institutions.

In conclusion, we have built a national cohort of gastric ER in Korea using the NHIS database. Our study demonstrates a tendency for increasing numbers of gastric ESD. There was a significant variance in the number of ER procedures by institutional type or procedural volume, and most operations were concentrated in a small number of large-volume centers. Differences in the distribution of types, regions, and medical resources according to the procedural volume were also demonstrated. This study will subsequently serve as a foundation for understanding the current status of gastric ER and the distribution of medical resources more accurately on a national scale, which could be useful for academic purposes such as establishing a long-term nationwide registry for gastric ER.

## Supplementary Information

Supplementary Tables.

## Data Availability

All data generated or analyzed during this study are included in this published article and its Supplementary Information files. The data presented in this study are available from the corresponding author on reasonable request.

## References

[1] Kim SG (2019). Current status of endoscopic submucosal dissection for early gastric cancer in Korea: Role and benefits. Korean J. Intern. Med..

[2] Hahn KY (2018). Comparative study between endoscopic submucosal dissection and surgery in patients with early gastric cancer. Surg. Endosc..

[3] Shin DW, Hwang HY, Jeon SW (2017). Comparison of endoscopic submucosal dissection and surgery for differentiated type early gastric cancer within the expanded criteria. Clin. Endosc..

[4] Park CH (2020). Clinical practice guideline for endoscopic resection of early gastrointestinal cancer. Clin. Endosc..

[5] Pimentel-Nunes P (2019). Management of epithelial precancerous conditions and lesions in the stomach (MAPS II): European Society of Gastrointestinal Endoscopy (ESGE), European Helicobacter and Microbiota Study Group (EHMSG), European Society of Pathology (ESP), and Sociedade Portuguesa de Endoscopia Digestiva (SPED) guideline update 2019. Endoscopy.

[6] Banks M (2019). British Society of Gastroenterology guidelines on the diagnosis and management of patients at risk of gastric adenocarcinoma. Gut.

[7] Lee EH (2011). Trends in Cancer Screening Rates among Korean Men and Women: Results from the Korean National Cancer Screening Survey (KNCSS), 2004–2010. Cancer Res. Treat..

[8] Odagiri H (2017). Hospital volume and adverse events following esophageal endoscopic submucosal dissection in Japan. Endoscopy.

[9] Jun JK (2017). Effectiveness of the Korean National Cancer Screening Program in reducing gastric cancer mortality. Gastroenterology.

[10] Gambitta P (2018). Endoscopic submucosal dissection versus endoscopic mucosal resection for type 0-II superficial gastric lesions larger than 20 mm. Ann. Gastroenterol..

[11] Tanabe S (2014). Long-term outcomes of endoscopic submucosal dissection for early gastric cancer: A retrospective comparison with conventional endoscopic resection in a single center. Gastric Cancer.

[12] Kim SG (2018). Long-term clinical outcomes of endoscopic submucosal dissection in patients with early gastric cancer: A prospective multicenter cohort study. Gut Liver..

[13] Cao Y (2009). Meta-analysis of endoscopic submucosal dissection versus endoscopic mucosal resection for tumors of the gastrointestinal tract. Endoscopy.

[14] Lee SB, Kang HY, Kim KI, Ahn DH (2010). The diagnostic accuracy of endoscopic biopsy for gastric dysplasia. J. Gastric Cancer..

[15] Choi CW (2014). The risk factors for discrepancy after endoscopic submucosal dissection of gastric category 3 lesion (low grade dysplasia). Dig. Dis. Sci..

[16] Ryu JE (2022). Trends in the performance of the Korean National Cancer Screening Program for gastric cancer from 2007 to 2016. Cancer Res. Treat..

[17] Park SH, Kang MJ, Yun EH, Jung KW (2022). Epidemiology of Gastric Cancer in Korea: Trends in incidence and survival based on Korea Central Cancer Registry Data (1999–2019). J. Gastric Cancer..

[18] Information Committee of the Korean Gastric Cancer Association (2021). Korean Gastric Cancer Association-Led Nationwide Survey on surgically treated gastric cancers in 2019. J. Gastric Cancer..

[19] The results of the 1st adequacy evaluation of stomach cancer. Health Insurance Review and Assessment Service. 2016. https://www.hira.or.kr/ra/eval/asmWrptPopup.do?evlCd=24&pgmid=HIRAA030004000000. Accessed 02 April 2023.

[20] The results of the 4th adequacy evaluation of stomach cancer. Health Insurance Review and Assessment Service. 2019. https://www.hira.or.kr/ra/eval/asmWrptPopup.do?evlCd=24&pgmid=HIRAA030004000000. Accessed 02 April 2023.

[21] Park CH (2013). Long-term outcome of early gastric cancer after endoscopic submucosal dissection: Expanded indication is comparable to absolute indication. Dig. Liver Dis..

[22] Kang MS (2015). Long-term outcome after endoscopic submucosal dissection for early gastric cancer: Focusing on a group beyond the expanded indication. J. Dig. Dis..

[23] Cho S, Chang Y, Kim Y (2019). Cancer patients' utilization of tertiary hospitals in Seoul Before and after the benefit expansion policy. J. Prev. Med. Public Health..

[24] Komatsu K, Teraura H, Yamaguchi H, Kotani K (2019). Difference in the types of treated cancer between clinics and small-to-middle-sized hospitals in rural communities of Japan. Tohoku J. Exp. Med..

[25] Laconi E, Marongiu F, DeGregori J (2020). Cancer as a disease of old age: Changing mutational and microenvironmental landscapes. Br. J. Cancer..

[26] Woo EK (2007). Morbidity and related factors among elderly people in South Korea: Results from the Ansan Geriatric (AGE) cohort study. BMC Public Health.

[27] Baek JY, Lee E, Jung HW, Jang IY (2021). Geriatrics fact sheet in Korea 2021. Ann. Geriatric Med. Res..

[28] Oda I, Suzuki H, Nonaka S, Yoshinaga S (2013). Complications of gastric endoscopic submucosal dissection. Dig. Endosc..

[29] Matsui N, Akahoshi K, Nakamura K, Ihara E, Kita H (2012). Endoscopic submucosal dissection for removal of superficial gastrointestinal neoplasms: A technical review. World J. Gastrointest. Endosc..

[30] Saito I (2014). Complications related to gastric endoscopic submucosal dissection and their managements. Clin. Endosc..

