# Supplementary material for: A 6-year nationwide population-based study on the current status of gastric endoscopic resection in Korea using administrative data
Source: Sci Rep. 2023 May 3;13:7203. doi: 10.1038/s41598-023-34215-7 (PMC10156708; doi:10.1038/s41598-023-34215-7)
Supplement: Supplementary file 1 — Supplementary Tables. [file 41598_2023_34215_MOESM1_ESM.pdf]

Title: A 6-year nationwide population-based study on the current status of gastric endoscopic resection in Korea using administrative data

Journal: Scientific Reports

Authors: Jae Yong Park, Mi-Sook Kim, Beom Jin Kim, Jae Gyu Kim*

Correspondence:

Jae Gyu Kim, M.D., Ph.D.

Department of Internal Medicine, Chung-Ang University College of Medicine, Seoul, Republic of Korea.

E-mail: jgkimd@cau.ac.kr

Supplementary Table 1. Annual numbers of gastric endoscopic resection during the study period

Year	Gastric cancer			Gastric adenoma		
	Total	ESD	EMR	Total	ESD	EMR
2012	9,475	7,502	1,973	16,045	8,021	8,024
2013	10,766	8,508	2,258	17,214	8,924	8,290
2014	11,375	8,952	2,423	17,465	9,440	8,025
2015	11,402	9,112	2,290	17,425	9,732	7,693
2016	12,625	10,205	2,420	19,186	11,174	8,012
2017	13,070	10,685	2,385	19,322	11,645	7,677
Total	68,713	54,964	13,749	106,657	58,936	47,721

ESD, endoscopic submucosal dissection; EMR, endoscopic mucosal resection.

Supplementary Table 2. The average annual number of ESD by type of medical institution

Average annual number of procedures (ESD)	Total (n=286)	Type of institution			
		Clinic (n=22)	Hospital (n=46)	General hospital (n=175)	Tertiary hospital (n=43)
Mean ± SD	68.1 ± 133.1	1.9 ± 1.9	9.8 ± 10.8	38.3 ± 59.2	285.4 ± 218.0
Median (IQR)	13.3 (4.0, 64.5)	1.0 (1.0, 2.0)	6.7 (2.0, 11.0)	14.5 (5.0, 51.3)	221.2 (138.7, 336.2)
Min	1.0	1.0	1.0	1.0	49.0
Max	1176.5	7.5	46.2	412.7	1176.5

ESD, endoscopic submucosal dissection; SD, standard deviation; IQR, interquartile range.

Supplementary Table 3. The average annual number of EMR by type of medical institution

Average annual number of procedures (EMR)	Total (n=838)	Type of institution			
		Clinic (n=307)	Hospital (n=224)	General hospital (n=264)	Tertiary hospital (n=43)
Mean ± SD	13.5 ± 37.6	3.2 ± 7.3	3.4 ± 4.9	15.7 ± 26.2	125.8 ± 95.8
Median (IQR)	2.3 (1, 7.0)	1.5 (1, 2.7)	2.0 (1, 3.5)	6.0 (2.3, 17.5)	99.2 (60.7, 148.0)
Min	1.0	1.0	1.0	1.0	14.0
Max	464.7	89.3	40.0	224.67	464.7

EMR, endoscopic mucosal resection; SD, standard deviation; IQR, interquartile range.

Supplementary Table 4. The average annual number of ER for gastric cancer by type of medical institution

Average annual number of procedures	Total (n=298)	Type of institution			
		Clinic (n=24)	Hospital (n=53)	General hospital (n=178)	Tertiary hospital (n=43)
Mean ± SD	38.8 ± 97.0	0.9 ± 0.8	2.1 ± 2.6	18.6 ± 40.8	189.1 ± 179.3
Median (IQR)	2.6 (0.8, 25.3)	0.8 (0.5, 1.0)	1.0 (0.4, 2.5)	2.8 (1.0, 15.2)	137.5 (90.2, 216.0)
Min	0.2	0.2	0.2	0.2	34.2
Max	960.5	3.8	12.2	319.5	960.5

ER, endoscopic resection; SD, standard deviation; IQR, interquartile range.

Supplementary Table 5. The average annual number of ER for gastric adenoma by type of medical institution

Average annual number of procedures	Total (n=846)	Type of institution			
		Clinic (n=315)	Hospital (n=223)	General hospital (n=265)	Tertiary hospital (n=43)
Mean ± SD	22.5 ± 60.7	3.2 ± 7.1	4.8 ± 8.1	27.9 ± 44.2	222.1 ± 125.4
Median (IQR)	2.5 (1, 10.3)	1.5 (1.0, 2.7)	2.0 (1.0, 4.0)	10.2 (3.0, 33.7)	199.7 (141.2, 258.0)
Min	0.5	0.5	0.5	0.5	11.3
Max	680.7	87.8	49.3	317.8	680.7

ER, endoscopic resection; SD, standard deviation; IQR, interquartile range.

Supplementary Table 6. The average annual number of ESD for gastric cancer by type of medical institution

Average annual number of procedures	Total (n=224)	Type of institution			
		Clinic (n=4)	Hospital (n=29)	General hospital (n=148)	Tertiary hospital (n=43)
Mean ± SD	41.2 ± 89.3	1.0 ± 1.0	2.5 ± 2.5	17.8 ± 35.5	151.7 ± 149.4
Median (IQR)	5.9 (1.2, 42.5)	0.8 (0.3, 1.7)	1.6 (0.5, 4.3)	4.4 (1.0, 16.3)	115.7 (65.7, 178.0)
Min	0.2	0.2	0.2	0.2	25.8
Max	796.2	2.4	9.0	256.8	796.2

ESD, endoscopic submucosal dissection; SD, standard deviation; IQR, interquartile range.

Supplementary Table 7. The average annual number of ESD for gastric adenoma by type of medical institution

Average annual number of procedures	Total (n=279)	Type of institution			
		Clinic (n=20)	Hospital (n=44)	General hospital (n=172)	Tertiary hospital (n=43)
Mean ± SD	36.2 ± 58.8	1.6 ± 1.6	8.0 ± 9.6	23.0 ± 30.8	133.7 ± 83.9
Median (IQR)	10.3 (2.3, 41.2)	1.0 (1.0, 1.8)	4.8 (1.1, 10.0)	10.8 (3.1, 29.8)	129.0 (75.8, 176.7)
Min	0.2	0.2	0.3	0.2	7.0
Max	380.3	6.5	40.3	193.2	380.3

ESD, endoscopic submucosal dissection; SD, standard deviation; IQR, interquartile range.

Supplementary Table 8. The average annual number of EMR for gastric cancer by type of medical institution

Average annual number of procedures	Total (n=260)	Type of institution			
		Clinic (n=22)	Hospital (n=44)	General hospital (n=151)	Tertiary hospital (n=43)
Mean ± SD	9.0 ± 19.5	0.8 ± 0.5	0.8 ± 0.8	4.5 ± 8.6	37.3 ± 32.9
Median (IQR)	1.1 (0.5, 9.6)	0.8 (0.5, 1.0)	0.5 (0.3, 1.0)	1.0 (0.4, 4.3)	24.5 (18.0, 46.7)
Min	0.2	0.2	0.2	0.2	3.5
Max	164.3	2.0	3.3	62.7	164.3

EMR, endoscopic mucosal resection; SD, standard deviation; IQR, interquartile range.

Supplementary Table 9. The average annual number of EMR for gastric adenoma by type of medical institution

Average annual number of procedures	Total (n=829)	Type of institution			
		Clinic (n=302)	Hospital (n=220)	General hospital (n=264)	Tertiary hospital (n=43)
Mean ± SD	10.8 ± 28.2	3.2 ± 7.2	3.3 ± 4.6	13.0 ± 20.7	88.4 ± 74.7
Median (IQR)	2.0 (1.0, 6.7)	1.5 (1.0, 2.7)	2.0 (1.0, 3.5)	5.2 (2.0, 14.3)	63.8 (40.7, 108.2)
Min	0.3	0.5	0.3	0.3	4.3
Max	345.2	87.8	36.8	162.0	345.2

EMR, endoscopic mucosal resection; SD, standard deviation; IQR, interquartile range.

Supplementary Table 10. The average annual number of EMR by procedural volume

Average annual number of procedures	Total (n=838)	Procedural volume of EMR			
		Very low (n=678)	Low (n=117)	High (n=30)	Very high (n=13)
Mean ± SD	13.5 ± 37.6	2.7 ± 2.2	28.4 ± 16.3	101.7 ± 24.9	240.2 ± 102.7
Median (IQR)	2.3 (1.0, 7.0)	2.0 (1.0, 3.5)	23.3 (14.7, 41.2)	97.3 (81.8, 127.0)	189.7 (177.2, 272.2)
Min	1.0	1.0	10.0	68.2	148.0
Max	464.7	9.8	89.3	147.3	464.7

EMR, endoscopic mucosal resection; SD, standard deviation; IQR, interquartile range.

Supplementary Table 11. Distribution of institutional type according to the classification by procedural volume of EMR

Type of institution	Procedural volume of EMR			
	Very low (n=678)	Low (n=117)	High (n=30)	Very high (n=13)
Clinics	293 (43.2%)	14 (12.0%)	0 (0.0%)	0 (0.0%)
Hospitals	210 (31.0%)	14 (12.0%)	0 (0.0%)	0 (0.0%)
General hospitals	175 (25.8%)	76 (65.0%)	11 (36.7%)	2 (15.4%)
Tertiary hospitals	0 (0.0%)	13 (11.1%)	19 (63.3%)	11 (84.6%)

EMR, endoscopic mucosal resection.

Supplementary Table 12. Regional distribution of institutions according to the classification by procedural volume of EMR

Region	Procedural volume of EMR			
	Very low (n=678)	Low (n=117)	High (n=30)	Very high (n=13)
The Seoul Capital Area	270 (39.8)	49 (41.9)	16 (53.3)	4 (30.8)
Metropolitan cities	165 (24.3)	31 (26.5)	7 (23.3)	8 (61.5)
Provinces	243 (35.8)	37 (31.6)	7 (23.3)	1 (7.7)

EMR, endoscopic mucosal resection.

Supplementary Table 13. Concordance between the patients' residence and the location of institutions where they underwent ESD

(concordance test results: p<.001*)

Location of institutions	Patients' residence			Total[a]
	The Seoul Capital Area	Metropolitan cities	Provinces	
The Seoul Capital Area	45,879	2,324	11,639	59,842
Metropolitan cities	217	16,903	9,946	27,066
Provinces	580	1,867	17,921	20,368
Total	46,676	21,094	39,506	107,276

* Bowker's test of symmetry was used to determine the concordance between the location of patients’ residence and the location of medical institution where they received ESD.

a. Frequency of missing values = 21

ESD, endoscopic submucosal dissection.

Supplementary Table 14. Patients' residence and the location of institutions where they underwent EMR

(concordance test results: p<.001*)

Location of institutions	Patients' residence			Total[a]
	The Seoul Capital Area	Metropolitan cities	Provinces	
The Seoul Capital Area	21,365	774	4,182	26,321
Metropolitan cities	206	15,314	6,687	22,207
Provinces	295	488	11,570	12,353
Total	21,866	16,576	22,439	60,881

* Bowker's test of symmetry was used to determine the concordance between the location of patients' residence and the location of medical institution where they received EMR.

a. Frequency of missing values = 9

EMR, endoscopic mucosal resection.

Supplementary Table 15. Distribution of medical resources according to the classification by procedural volume of EMR

Medical resources	Procedural volume of EMR			
	Very low (n=678)	Low (n=117)	High (n=30)	Very high (n=13)
Doctors	13.3 ± 19.2	125.3 ± 128.4	379.7 ± 252.6	606.3 ± 449.2
Specialists	12.1 ± 15.7	78.1 ± 65.5	210.3 ± 123.0	356.2 ± 266.0
Hospital beds	110.1 ± 122.1	379.6 ± 239.8	782.0 ± 228.3	1146.2 ± 577.4
Operating rooms	1.6 ± 2.0	6.9 ± 5.6	15.5 ± 8.9	28.7 ± 21.8

Values are mean ± standard deviation unless stated otherwise. EMR, endoscopic mucosal resection.
